# Intravitreal Bevacizumab for Choroidal Neovascularization Secondary to Non-Age-Related Macular Degeneration

**Published:** 2010-01

**Authors:** Masoud Salehipour, Nasser Vafi, Azade Doozande, Mehdi Yaseri

**Affiliations:** 1 Shahid Mohammadi Hospital, Bandar Abbas, Iran; 2 Negah Eye Center, Tehran, Iran; 3 Labbafinejad Medical Center, Shahid Beheshti University of Medical Sciences, Tehran, Iran

**Keywords:** Bevacizumab, Choroidal Neovascularization, Non-Age-Related Macular Degeneration

## Abstract

**Purpose:**

To report the long-term results of intravitreal bevacizumab (Avastin) therapy for choroidal neovascularization (CNV) secondary to non-age-related macular degeneration (non-AMD).

**Methods:**

This prospective interventional case series was conducted on patients with non-AMD CNV. All patients received 1.25 mg intravitreal bevacizumab and were followed for at least 18 weeks. Indications for retreatment were decreased visual acuity or recurrence of subretinal fluid or hemorrhage associated with leakage on fluorescein angiography. Primary outcome measures were changes in best-corrected visual acuity (BCVA) and central macular thickness (CMT). Secondary outcome measures consisted of any adverse event related to the therapy.

**Results:**

The study included 31 eyes of 28 patients with non-AMD CNV including idiopathic (n=11), due to myopia (n=7), angioid streaks (n=5), and other disorders (n=8). Mean initial BCVA was 20/100 which improved to 20/60 at 6 weeks; 20/40 at 12, 18, 24, and 36 weeks; and 20/30 at 54 weeks. Serial optical coherence tomography measurements showed mean CMT of 288 μm at baseline, which was decreased to 209 μm at last visit (P=0.95). There was no correlation between the underlying disease and changes in BCVA during the follow-up period.

**Conclusion:**

Intravitreal bevacizumab significantly improved visual acuity in eyes with non-AMD CNV due to various etiologies.

## INTRODUCTION

Choroidal neovascularization (CNV) may develop in the course of over 30 different ocular disorders.^1^,^2^ The most common cause of CNV apart from age-related macular degeneration (AMD) is pathologic myopia.^3^ In patients aged 50 years or younger, CNV may develop in the absence of any identifiable cause and is considered to be idiopathic.^3^,^4^ Other less common causes of CNV include angioid streaks, central serous chorioretinopathy (CSC), ocular histoplasmosis syndrome (OHS), multifocal choroiditis, trauma, and hereditary eye diseases.^3^,^5^,^6^

A number of studies have demonstrated that the vascular endothelial growth factor (VEGF), a potent mitogen for endothelial cells, plays a principal role in the development of neovascularization in various chorioretinal diseases.^7^,^8^ There have been recent reports on the use of intravitreal bevacizumab (IVB), a pan anti-VEGF antibody, for CNV secondary to high myopia,^9^–^20^ angioid streaks,^21^,^22^ CSC,^23^ idiopathic,^23^,^24^ OHS,^25^,^26^ and choroidal osteoma.^27^ However, limited information is available on the long-term effect of treatment with VEGF-blocking agents on CNV secondary to non-AMD causes. Herein we present the long-term outcomes of IVB therapy on non-AMD CNV due to various etiologies.

## METHODS

This prospective interventional case series includes consecutive patients with non-AMD CNV who were recruited from the Department of Ophthalmology at Labbafinejad Medical Center, a tertiary referral eye care center in Tehran. Inclusion criteria were: clinical setting suggesting CNV of non-AMD origin, subfoveal or juxtafoveal location of the lesion and evidence of leakage on fluorescein angiography (FA). Exclusion criteria consisted of clinical features suggesting CNV secondary to AMD, diabetic retinopathy and glaucoma. However, patients who had previously received thermal laser therapy or photodynamic therapy (PDT) for CNV were not excluded. The Ethics Committee of the Ophthalmic Research Center approved the study protocol, and informed consent regarding the risks, benefits, and alternatives of therapy was obtained from all patients.

At baseline, all patients underwent a comprehensive examination, including determination of best-corrected visual acuity (BCVA), slitlamp biomicroscopy, intraocular pressure (IOP) measurement, dilated fundus examination, color fundus photography, fluorescein angiography (FA) and optical coherence tomography (OCT). BCVA was evaluated by certified optometrists using a Snellen chart at 6m with conversion to logMAR equivalent for statistical analysis. OCT was performed by a technician using the three dimensional OCT-1000 (Topcon, Europe Medical B.V., Capelle a/d IJssel, The Netherlands), and central macular thickness (CMT) was measured using the retinal thickness map.

The decision for IVB injection was made according to presence of active CNV judged by the presence of leakage on FA, and intra- or subretinal fluid on OCT. Intravitreal bevacizumab injection was performed as an outpatient procedure under aseptic conditions in the operating room. Topical anesthesia was achieved using 0.5% tetracaine drops, and 5% povidone-iodine solution was applied to the eyelids and the cul-de-sac, followed by draping and insertion of a lid speculum. Intravitreal injection of 1.25 mg/0.05 ml bevacizumab (Avastin, Roche, Basel, Switzerland) was performed using a 30-gauge needle 4-mm posterior to the limbus in the superior temporal quadrant. Topical ciprofloxacin (4 times a day) was prescribed for 3 days after the injection.

Patients were examined the day after the procedure and after 1, 6, 12, 18, 24, 36, and 54 weeks. At each visit BCVA, a complete ocular examination and OCT were repeated; fundus photography and FA were performed as necessary. After initial treatment, the decision to retreat was based on clinical, OCT and angiographic findings. Subsequent injections were administered at least 6 weeks after a previous injection if the patients complained of decreased vision and/or increased metamorphopsia, or in cases which recurrence of intra- or subretinal fluid, or hemorrhage was associated with leakage on FA. Primary outcome measures were changes in BCVA and CMT based on OCT. Secondary outcome measures were any ocular or systemic adverse events during the study period.

Statistical analysis was performed using SPSS version 15.0 (SPSS Inc., Chicago, USA). Serial changes in BCVA and OCT were compared using paired *t*-test. Analysis of variance (ANOVA) was employed to compare changes in BCVA and the number of injections in relation to the underlying disease. P values less than 0.05 were considered as statistically significant.

## RESULTS

Thirty-one phakic eyes of 28 patients including 10 male and 18 female subjects with mean age of 37.8±12.1 (range, 11–67) years were enrolled. Patients’ characteristics are shown in Table 1. Underlying diagnoses associated with non- AMD CNV were idiopathic in 11 eyes, myopia in 7 eyes, angioid streaks in 5 eyes, and other causes in 8 eyes (ocular toxoplasmosis in 3 eyes, multifocal choroiditis in 2 eyes; and OHS, CSC and choroidal osteoma, each in 1 eye). Nine eyes had received 1 to 3 sessions of PDT 3 to 6 months prior to IVB injection, and one eye had 2 sessions of thermal laser therapy previously. IVB was used as primary treatment in the remaining 21 eyes. Mean follow-up period was 40.4±12 (range, 18–54) weeks.

**Figure 1:**
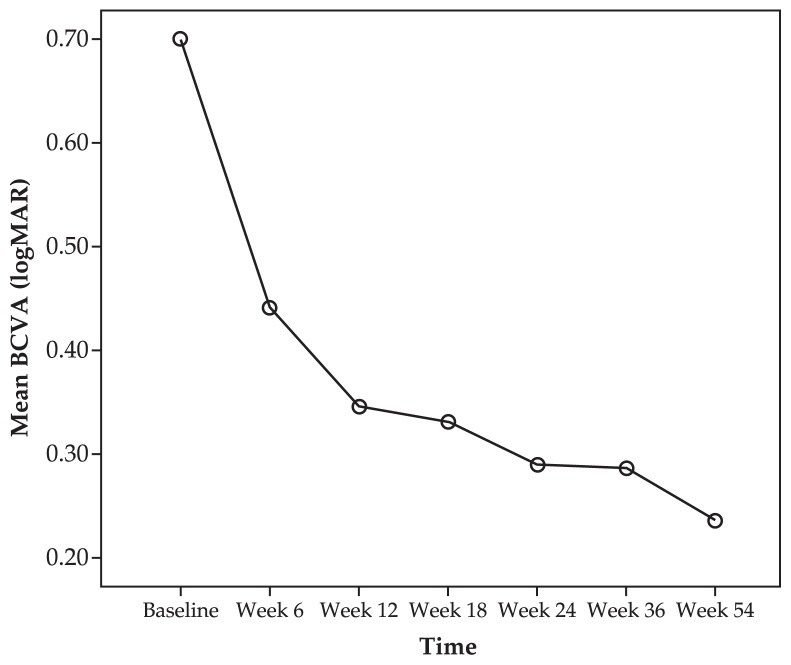
Changes in best-corrected visual acuity (BCVA) following intravitreal bevacizumab for treatment of non-age-related macular degeneration choroidal neovascularization.

Visual results are shown in Table 2 and outcomes within each subgroup are detailed in Table 3. Figure 1 shows changes in mean BCVA during the study period. Mean BCVA at baseline was 0.69±0.51 logMAR (20/100) which improved to 0.44±0.39 logMAR (20/60, P=0.001) 6 weeks after initial IVB injection and remained stable at 12 weeks (0.34±0.39 logMAR, 20/40, P<0.001), 18 weeks (0.33±0.37 logMAR, 20/40, P<0.001), 24 weeks (0.29±0.37 logMAR, 20/40, P<0.001), 36 weeks (0.28±0.32, 20/40, P=0.001), and final follow-up (0.23±0.26 logMAR, 20/30, P=0.029). Only one eye experienced BCVA deterioration ≥2 line from baseline at last follow-up. At week 54, 60% of eyes demonstrated visual improvement ≥2 lines, and pre-treatment BCVA was preserved in the remaining 40% (Table 2). Changes in BCVA during the study period showed no significant relationship with the underlying condition (P=0.14 and P=0.15 at 36 and 54 weeks, respectively). The mean number of intravitreal injections was 2.16 which also showed no correlation with the underlying disorder (P=0.19).

**Figure 2:**
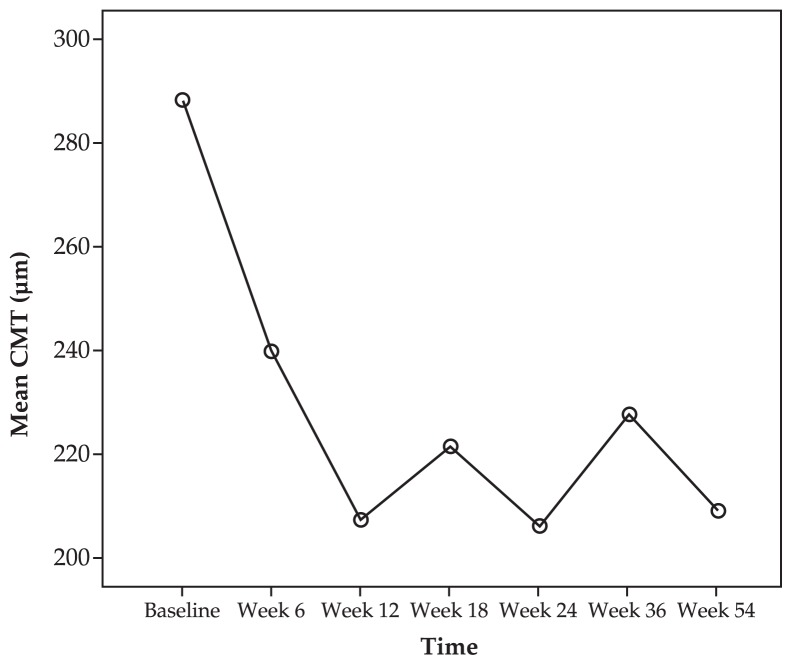
Changes in central macular thickness following intravitreal bevacizumab injection for treatment of non-age-related macular degeneration choroidal neovascularization.

**Figure 3:**
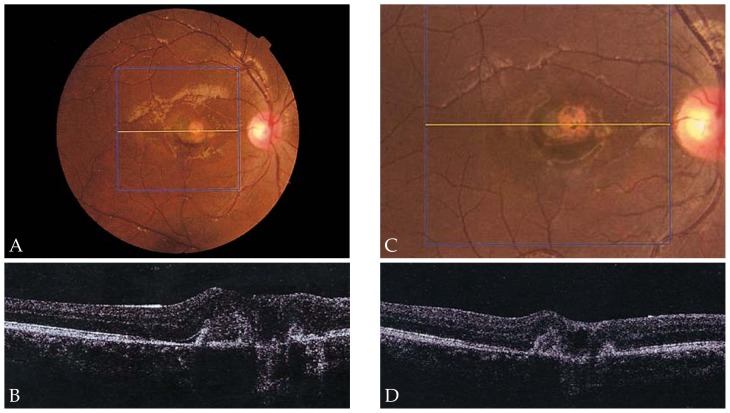
Fundus photograph **(A)** of a patient with choroidal neovascularization (CNV) and subretinal hemorrhage secondary to ocular toxoplasmosis with best-corrected visual acuity (BCVA) of 20/100 at baseline; central macular thickness (CMT) was 357 μm on optical coherence tomography (OCT) **(B)**. After 6 weeks, resolution of the subretinal hemorrhage is noted **(C)** with improvement of BCVA to 20/40; CMT is reduced to 276 μm **(D)**.

**Figure 4:**
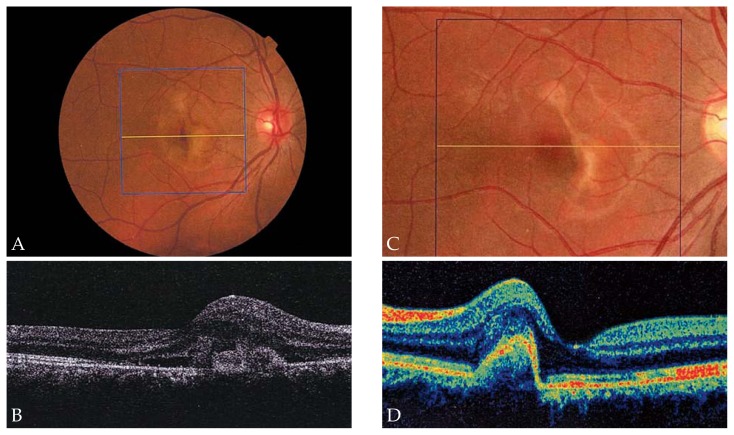
Fundus photograph and optical coherence tomography (OCT) of the right eye of a patient with choroidal neovascularization (CNV) secondary to central serous chorioretinopathy before and 3 months after a single intravitreal bevacizumab (IVB) injection. Fundus photograph demonstrates a juxtafoveal CNV with retinal hemorrhage around the CNV **(A)**, best-corrected visual acuity (BCVA) is 20/80. OCT shows macular thickening with intraretinal fluid and shallow pigment epithelial detachment **(B)**. Fundus photograph **(C)** and OCT **(D)** three months after IVB demonstrate resolution of the hemorrhage with scarring while BCVA improved to 20/25.

**Figure 5:**
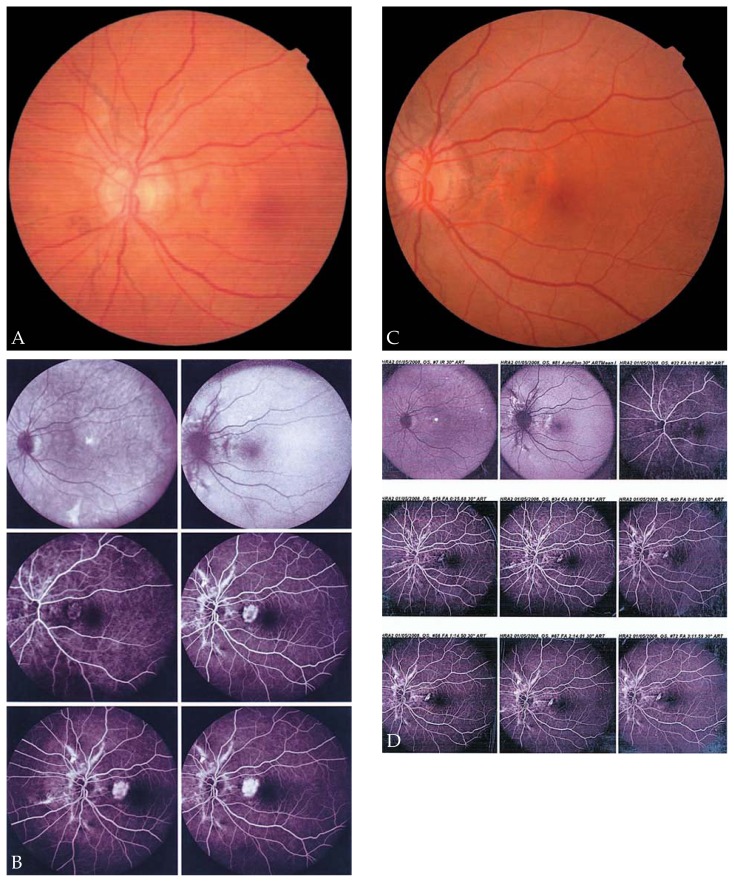
A 57-year-old woman with angioid streaks and prior history of photodynamic therapy, presented with metamorphopsia in the left eye while visual acuity decreased to 20/25. An extrafoveal choroidal neovascularization (CNV) with retinal hemorrhage and thickening was seen in the fundus **(A)**. The angiogram shows leakage in association with the recurrent CNV **(B)**. Three months after a single intravitreal bevacizumab (IVB) injection, resolution of thickening and hemorrhage is noted **(C)**. Angiography 3 months after IVB demonstrates resolution of leakage **(D)**, visual acuity improved to 20/20 without metamorphopsia.

**Figure 6:**
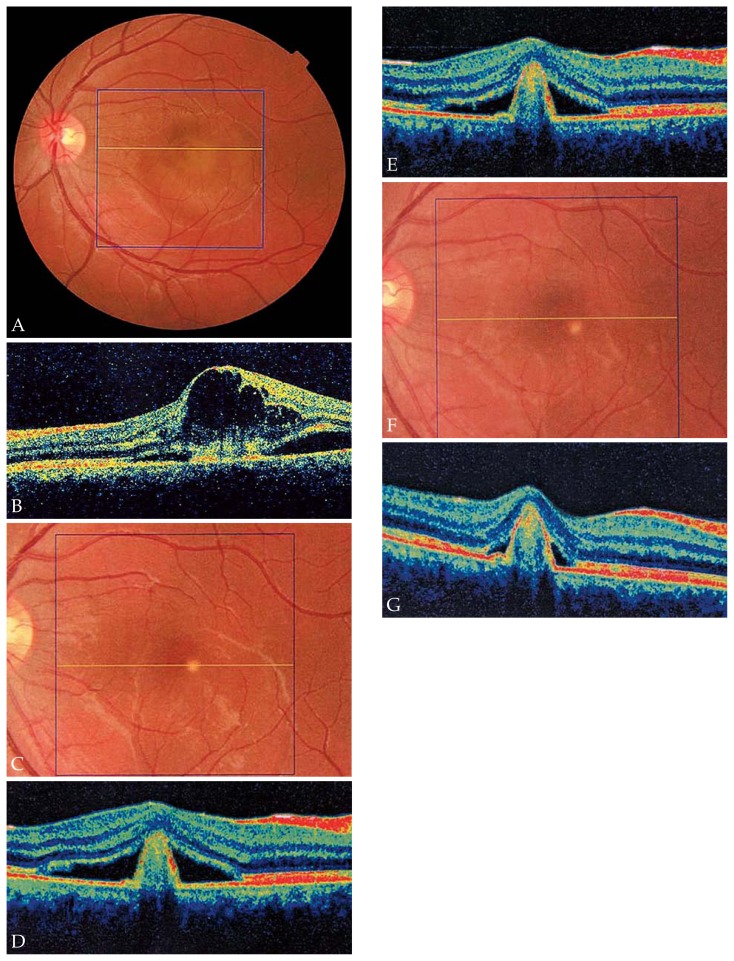
Fundus photography and optical coherence tomography (OCT) of the left eye of a patient with idiopathic choroidal neovascularization (CNV). A subfoveal CNV with subretinal fluid and retinal hemorrhage is noted **(A)**, baseline best-corrected visual acuity (BCVA) was 20/200. OCT demonstrates severe subretinal fluid with cystic spaces, and central macular thickness (CMT) of 598 μm **(B)**. After 6 months and 2 intravitreal bevacizumab (IVB) injections marked improvement is evident **(C)** and OCT shows resolution of cystic spaces with mild neurosensory detachment and CMT of 302 μm **(D)**. After 10 months, CMT reached 290 μm **(E)**. At final follow-up (after 13 months), the macula appeared normal, with a yellow subretinal spot **(F)**, and OCT shows absence of any intra- and sub-retinal fluid **(G)**. CMT and BCVA were 248 μm and 20/30, respectively.

Figure 2 shows serial changes in mean CMT during the study period. Mean CMT was 288 μm at baseline, decreased to 240 μm after 6 weeks, and was further reduced to 209 μm after 54 weeks. However, changes in CMT were not statistically significant (P= 0.95). Representative examples demonstrate the effect of IVB for treatment of CNV secondary to ocular toxoplasmosis (Fig. 3), CSC (Fig. 4), and angioid streaks (Fig. 5), and also for idiopathic CNV (Fig. 6).

None of the patients developed systemic (thromboembolic events or cerebral vascular accidents), or ocular (intraocular inflammation, IOP rise, cataract formation, endophthalmitis and retinal detachment) complications related to IVB injection.

## DISCUSSION

In this prospective study, eyes treated with IVB for non-AMD CNV showed significant visual improvement which was maintained for about one year. The current work may support the notion that VEGF is a critical stimulus for CNV formation in different clinical settings such as myopia, angioid streaks, toxoplasmosis, multifocal choroiditis, choroidal osteoma and ocular histoplasmosis. Based on data from the last follow-up, BCVA improved ≥2 lines in 60% of IVB treated eyes. These functional results are in agreement with a previous case series of IVB for non-AMD CNV, including idiopathic CNV (improvement in 50% of cases at 3 months^24^ and 73% of eyes at 6 months^23^), and CNV secondary to CSC and punctate inner choroidopathy (improvement in 73% of eyes at 6 months)^23^ where similar criteria for visual improvement were applied.

Visual results in our patients compare favorably with the natural history and with outcomes of previous treatment modalities for non-AMD CNV. Previous studies have shown with observation alone and with PDT, 42%^4^ and 58%^28^ of eyes with idiopathic CNV demonstrate ≥2 lines of visual acuity improvement. In contrast, in the present study, 75% of eyes in the subgroup with idiopathic CNV showed ≥2 lines of improvement following IVB injections.

Patients with CNV secondary to pathologic myopia have a poor natural course;^29^ in a randomized trial, eyes treated with PDT did not fare better than untreated controls.^30^ The effectiveness of IVB for myopic CNV has been evaluated in multiple case series.^9^–^20^ In a prospective, noncomparative interventional cases series, Ruiz-Moreno et al^17^ reported the one year results of IVB for treatment of subfoveal CNV in highly myopic eyes. Twenty-nine eyes underwent 3 monthly injections of 1.25 mg IVB and were evaluated in terms of BCVA and OCT at baseline and then monthly for one year. FA was performed at baseline, after 3 months and whenever CNV activity was suspected; this was similar to our treatment and follow-up protocol. Of 29 eyes, 16 were naive for treatment but 13 eyes had been previously treated with multiple sessions of PDT. Six eyes required repeat injections. Mean BCVA at baseline was 0.55 logMAR which improved to 0.38 logMAR at 1 year; CMT decreased significantly at month 12 as compared to baseline. The one year results of IVB injections (2.5 mg) for myopic CNV were also evaluated in a recent study on eyes without earlier treatment.^20^ Significant visual improvement and CMT reduction was observed in this series with a mean number of 1.4 injections within 12 months. The mean number of injections was 2 in our study with greater follow-up as compared to previous studies. Two other recent studies on IVB for myopic CNV have also demonstrated comparable results at one year.^18^,^19^

The visual prognosis of CNV secondary to angioid streaks is dismal; a group of untreated patients lost 6 lines of visual acuity in 18 months.^31^ The results of treatment for CNV secondary to angioid streaks are poor with thermal laser and disappointing with PDT. The visual results after PDT vary widely; however, all studies show loss of VA, including 1-line loss at 1-year,^32^ 4.9-line loss at 18 months^33^ and 3-line loss at 42 months.^34^ Therefore it might be inferred that PDT is not a good treatment modality for CNV secondary to angioid streaks.^31^,^32^,^34^ In contrast, IVB may lead to modest improvement or stabilization in vision and lesion size in such eyes.^22^,^33^,^35^,^36^ In the present study, none of the patients with angioid streaks lost VA until final follow-up; 20% demonstrated visual improvement ≥ 2 lines, and 80% maintained their pre-injection VA.

Schadlu et al^25^ reported the results of IVB in eyes with CNV resulting from OHS. Of 28 eyes, 20 (71%) experienced visual improvement, VA remained unchanged in 4 (14%) eyes, and 4 eyes experienced a decrease in vision. Mean follow-up was 22 weeks with an average of 1.8 intravitreal injections. Adan et al^26^ also reported a case of presumed ocular histoplasmosis which responded remarkably well to IVB. We had only one case of CNV secondary to presumed ocular histoplasmosis in our series. Marked visual improvement and resolution of metamorphopsia was noted in this patient after IVB injection.

The result of IVB in eyes with CNV secondary to multifocal choroiditis in our series were comparable to the report by Fine et al^37^ who noted improved vision in 5 of 6 eyes. Deterioration of VA occurred in one patient due to subfovel rip of the RPE. We have reported the results of treatment of CNV secondary to choroidal osteoma elsewhere.^27^

In our study, changes in VA and the number of injections were not correlated with the underlying conditions which is consistent with the report by Chang et al.^38^ In summary, the results of this prospective study suggest that anatomical and visual outcomes of IVB treatment for non-AMD CNV are favorable. Limitations of the study include the small number of cases and lack of a control group. However, the low prevalence of certain underlying conditions may prevent performing large, randomized, controlled trials similar to those conducted for CNV secondary to AMD. In addition, it would unethical to withhold treatment from patients with non-AMD CNV when the natural course of the condition is poor.

## Figures and Tables

**Table 1:** Clinical characteristics of patients enrolled in the study

Underlying diagnosis	No. of eyes	Mean age (years)	Male/Female	Mean follow-up (weeks)	Mean number of injections	History of PDT or thermal laser (No. of eyes)
Idiopathic	11	32.7	2/7	37.6	2.6	2
Myopia	7	44.4	2/5	46.2	2	5
Angioid streaks	5	51.4	2/3	39.6	2.6	2
Toxoplasmosis	3	24.7	2/1	50	1	
Multifocal choroiditis	2	30.3	Female	39.7	1.3	
Ocular histoplasmosis	1	47	Male	48	2	1
CSC	1	31	Male	24	1	
Choroidal osteoma	1	19	Female	30	2	
Total	31	37.7	10/18	40.4	2.1	10

No, number; PDT, photodynamic therapy; CSC, central serous chorioretinopathy

**Table 2:** Visual results of bevacizumab injection for non-AMD CNV

Visual parameter	Baseline	6 weeks	12 weeks	18 weeks	24 weeks	36 weeks	54 weeks
logMAR BCVA (Mean ± SD)	0.69±0.51	0.44±0.39	0.34±0.36	0.33±0.37	0.29±0.37	0.28±0.32	0.23±0.26

Mean Snellen BCVA	20/100	20/60	20/40	20/40	20/40	20/40	20/30

P-Value (compared to baseline)		0.001	<0.001	<0.001	<−0.001	0.001	0.029

BCVA changes ≥ 2 line							
Improved		44.0%	73.1%	63.6%	62.5%	63.2%	60.0%

Same		56.0%	23.1%	36.4%	37.5%	31.6%	40.0%

Worse		0.0%	3.8%	0.0%	0.0%	5.3%	0.0%

AMD, age-related macular degeneration; CVN, choroidal neovascularization; BCVA, best-corrected visual acuity; SD, standard deviation

**Table 3:** Snellen best corrected visual acuity (mean±SD) at baseline and after bevacizumab injection for subgroups of non-AMD CNV

Subgroup	Baseline	6 weeks	12 weeks	18 weeks	24 weeks	36 weeks
Idiopathic	0.58±0.53	0.47±0.042	0.35±35	0.32±0.37	0.28±0.41	0.26±0.33
	20/80	20/60	20/40	20/40	20/40	20/40

Myopia	1.07±0.55	0.44±0.22	0.57±0.33	0.79±0.27	0.52±0.45	0.40±0.34
	20/240	20/60	20/70	20/120	20/70	20/50

Angioid Streaks	0.40±0.54	0.47±0.55	0.28±0.42	0.21±0.41	0.05±0.70	0.21±0.37
	20/50	20/60	20/40	20/30	20/20	20/30

Others	0.72±0.22	0.36±0.44	0.20±0.33	0.17±0.17	0.20±0.28	0.33±0.21
	20/100	20/50	20/30	20/30	20/30	20/40
